# Double Auxotrophy to Improve the Safety of a Live Anti-*Pseudomonas aeruginosa* Vaccine

**DOI:** 10.3390/vaccines10101622

**Published:** 2022-09-27

**Authors:** Víctor Fuentes-Valverde, Patricia García, Miriam Moscoso, Germán Bou

**Affiliations:** 1 Department of Microbiology, University Hospital A Coruña (CHUAC), Biomedical Research Institute A Coruña (INIBIC), 15006 A Coruña, Spain; 2 Centro de Investigación Biomédica en Red de Enfermedades Infecciosas (CIBERINFEC), Instituto de Salud Carlos III, 28029 Madrid, Spain

**Keywords:** live-attenuated vaccine, *Pseudomonas aeruginosa*, auxotrophy, intranasal immunization, glutamate racemase gene, alanine racemase gene

## Abstract

*Pseudomonas aeruginosa* is an opportunistic nosocomial pathogen that causes serious infections in the respiratory tract of immunocompromised or critically ill patients, and it is also a significant source of bacteremia. Treatment of these infections can be complicated due to the emergence of multidrug-resistant *P. aeruginosa* strains worldwide. Hence, the development of prophylactic vaccines is a priority for at-risk patients. We have previously developed a vaccine candidate with a single auxotrophy for D-glutamate, PAO1 Δ*murI*, which protects against sepsis and acute pneumonia caused by *P. aeruginosa*. Given the paramount importance of safety in the development of live attenuated vaccines, we have improved the safety of the vaccine candidate by reducing the probability of a reversion to virulence by the inclusion of an additional auxotrophy for D-alanine. Single and double auxotrophs behaved in a similar manner in relation to the attenuation level, immunogenicity and protective efficacy, but the double auxotroph has the advantage of being more stable and safer as a candidate vaccine against respiratory infections caused by *P. aeruginosa*.

## 1. Introduction

*Pseudomonas aeruginosa* is regarded as an opportunistic pathogen that can cause acute healthcare-associated pneumonia in immunocompromised individuals, as well as chronic lung infection in patients with cystic fibrosis, bronchiectasis, or chronic obstructive pulmonary disease [1]. It is often associated with skin infections around burn wounds, urinary tract infections and serious bloodstream infections [2]. *P. aeruginosa* has become a major health concern due to its intrinsic resistance and the increasing multidrug resistance worldwide. To tackle this problem, vaccines could be used as an alternative to antibiotics. However, despite many efforts and some advances in pre-clinical studies, to date, there is no *P. aeruginosa* vaccine available for clinical use [3,4]. A successful vaccine against *P. aeruginosa* should elicit a combination of protective antibodies and a mixed Th1/Th2 or Th1/Th17 response [3,5]. Additionally, mucosal immunity generated via intranasal (IN) inoculation, which mimics the natural route of entry, will protect against *P. aeruginosa* respiratory infections [6]. We have already successfully used the PAO1 Δ*murI* strain, which lacks the *murI* (PA4662) gene encoding the glutamate racemase (EC 5.1.1.3), as a vaccine candidate [7]. This enzyme is responsible for the synthesis of D-glutamate, an essential component of peptidoglycan in bacteria. PAO1 Δ*murI* has been administered via the IN route in mice to generate a local immune response at mucosal surfaces of the lungs [8], and has demonstrated cross-protection against several *P. aeruginosa* strains [7]. Unfortunately, some prototrophic variants were detected over time and at a very high bacterial cell density. We now aim to improve the safety profile of the vaccine candidate by generating a strain that is also auxotrophic for D-alanine, a basic amino acid of bacterial cell wall taking part in the cross-links of glycan strands. For this purpose, the genes encoding the alanine racemases (EC 5.1.1.1) Alr and DadX, which are involved in the D-alanine biosynthesis of *P. aeruginosa* PAO1, were deleted [9]. We demonstrated that the new vaccine candidate PAO1 Δ*murI* Δ*alr* Δ*dadX* was able to provide protection against IN infection with strain PA14, a hypervirulent ExoU-producing *P. aeruginosa*. Interestingly, the toxin ExoU was present in 20% of clinical isolates and was associated with cytotoxicity, dissemination, lung epithelial injury, and a generally poor clinical outcome [1,10].

## 2. Materials and Methods

### 2.1. Genetic Manipulations

To produce the double auxotrophic derivative PAO1 Δ*murI* Δ*alr* Δ*dadX* (hereafter, PAO1 ΔΔΔ), unmarked in-frame deletions of *alr* (PA4930) and *dadX* (PA5302) genes in PAO1 Δ*murI* strain were generated using the pKNG101 allelic exchange system [11]. The primers used in this study are listed in Table 1. Briefly, upstream and downstream regions of *alr* and *dadX* genes were amplified from the *P. aeruginosa* PAO1 chromosome by PCR with specific primer pairs. The subsequent upstream and downstream fragments were digested with BamHI and NotI (*alr* upstream), NotI and SalI (*dadX* upstream) or NotI and ApaI (*alr* and *dadX* downstream). Digested products were then ligated into the linearized pKNG101 suicide vector. The resulting plasmids, pKNG101(UP-Δ*alr*-DN) and pKNG101(UP-Δ*dadX*-DN), were transformed in *Escherichia coli* SM10 λ*pir* by electroporation (with the following settings: 200 Ω, 18 kV/cm, 25 μF), before being plated on Luria-Bertani (LB) agar containing 50 μg/mL of streptomycin (Sm). Transformants were analyzed by PCR with *alr*-EXT R and *alr*-EXT F (or *dadX*-EXT R and *dadX*-EXT F) and positive clones were verified by sequencing. The recombinant plasmid pKNG101(UP-Δ*alr*-DN) was then conjugated into the PAO1 Δ*murI* strain by biparental mating as described elsewhere [12] and merodiploid colonies were selected on LB agar plates supplemented with 25 μg/mL chloramphenicol (Cm), 2000 μg/mL Sm and 10 mM D-glutamate. The first crossover event and thus the integration of the whole vector into the chromosome was confirmed by PCR amplification with RpKn and *alr*-EXT R primers. These cointegrates were grown in LB containing 15% sucrose, 10 mM D-glutamate and 10 mM D-alanine, and they were plated on the same media in order to select the second crossover event. The resultant colonies that grew on LB agar supplemented with 15% sucrose, 10 mM D-glutamate and 10 mM D-alanine, but that were sensitive on LB agar plates containing Cm (25 μg/mL), Sm (2000 μg/mL) and 10 mM D-glutamate, were then analyzed by PCR using *alr*-EXT F and *alr*-EXT R primers. The deletion mutant PAO1 Δ*murI* Δ*alr* was confirmed by sequence analysis. Similarly, the recombinant plasmid pKNG101(UP-Δ*dadX*-DN) was conjugated from *E. coli* SM10 λ*pir* into *P. aeruginosa* PAO1 Δ*murI* Δ*alr*, to generate the triple mutant PAO1 Δ*murI* Δ*alr* Δ*dadX*.

### 2.2. Growth, Viability and Phenotypic STABILITY

To assess their growth and viability, the PAO1 Δ*murI* and PAO1 ΔΔΔ strains were grown overnight at 37 °C with agitation in 5 mL of LB containing 8 mM D-glutamate or 8 mM D-glutamate plus 6 mM D-alanine, respectively. An aliquot of the suspension was then removed and used to inoculate 100 mL of LB supplemented or not supplemented with D-amino acids at an initial optical density at 600 nm (OD_600_) of 0.01. The inoculated suspensions were then incubated at 37 °C and 180 rpm. Samples were taken every hour up to 8 h to determine the culture turbidity (OD_600_) and the colony-forming units (CFU) on supplemented LB agar plates. At the same time, to determine the phenotypic stability, 10 mL aliquots were removed at 0, 4, 7 and 24 h, centrifuged (5000× *g*, 10 min, 4 °C) and washed thrice with phosphate-buffered saline (PBS) to remove excess D-amino acids, before plating on LB agar. After incubation of the plate at 37 °C for 48 h, the visible revertant colonies were transferred by streaking on a new LB agar plate to confirm prototrophy and were analyzed by PCR. To estimate the reversion frequency, overnight cultures of PAO1 ΔΔΔ were diluted (1:200) in 500 mL of LB supplemented with 8 mM D-glutamate and 6 mM D-alanine, and incubated at 37 °C, 180 rpm for 24 h. Cells were harvested by centrifugation, washed thrice and resuspended in 10 mL of PBS. The total volume was then spread on LB agar plates and the appearance of revertant colonies was examined as above. Additionally, the phenotypic stability of the double auxotrophic strain was tested for 5 days of incubation in supplemented medium and every 24 h, samples of the cultures (30 mL) were collected and spread on LB agar plates to examine the appearance of revertant colonies. All these assays were carried out in triplicate.

### 2.3. Animal Experiments

Mice were bred and maintained in the specific pathogen-free facility at the Centro Tecnológico de Formación de la Xerencia de Xestión Integrada A Coruña (CTF-XXIAC), Servicio Galego de Saúde (SERGAS). BALB/c mice at 10 to 12 weeks of age were used in all experiments.

For inoculations, bacteria were grown at 37 °C, 180 rpm until an OD_600_ of 0.7 was reached. The cells were then harvested by centrifugation (5000× *g*, 10 min, 4 °C), washed thrice and suspended in sterile saline solution (0.9% NaCl) to achieve the appropriate dose. The resultant suspension was administered by intraperitoneal (IP, 100 μL) injection or via the IN route (20 μL) to anaesthetized mice. Control mice were administered saline solution. Vaginal lavage fluid (VLF) and blood samples were collected by washing the vagina with sterile saline (50 µL) and by puncture of the submandibular vein, respectively. These biological samples were then centrifuged to separate them from cells (1500× *g*, 15 min, 4 °C) and kept at −80 °C until use.

### 2.4. ELISA

The levels of specific antibody IgG in mouse sera and of IgA in VLF were assessed by a whole-bacterial cell ELISA in accordance with the previously described protocol [7,8]. The secondary antibodies used were horseradish peroxidase (HRP)-conjugated anti-mouse IgG (Sigma-Aldrich) or IgA (Bethyl Laboratories) diluted 1:5000 in DMEM supplemented with 10% fetal bovine serum.

### 2.5. Statistical Analysis

The GraphPad Prism software package (version 6.01) was used for the statistical evaluation of the results. The Mann–Whitney U test was used to compare differences between two independent groups. Survival rates were compared using the log-rank Mantel–Cox test. Means were compared using an unpaired t-test, applied by the Holm–Sidak method.

## 3. Results

### 3.1. Construction and Characterization of the Double Auxotroph for D-Glutamate plus D-Alanine

**Figure 1:**
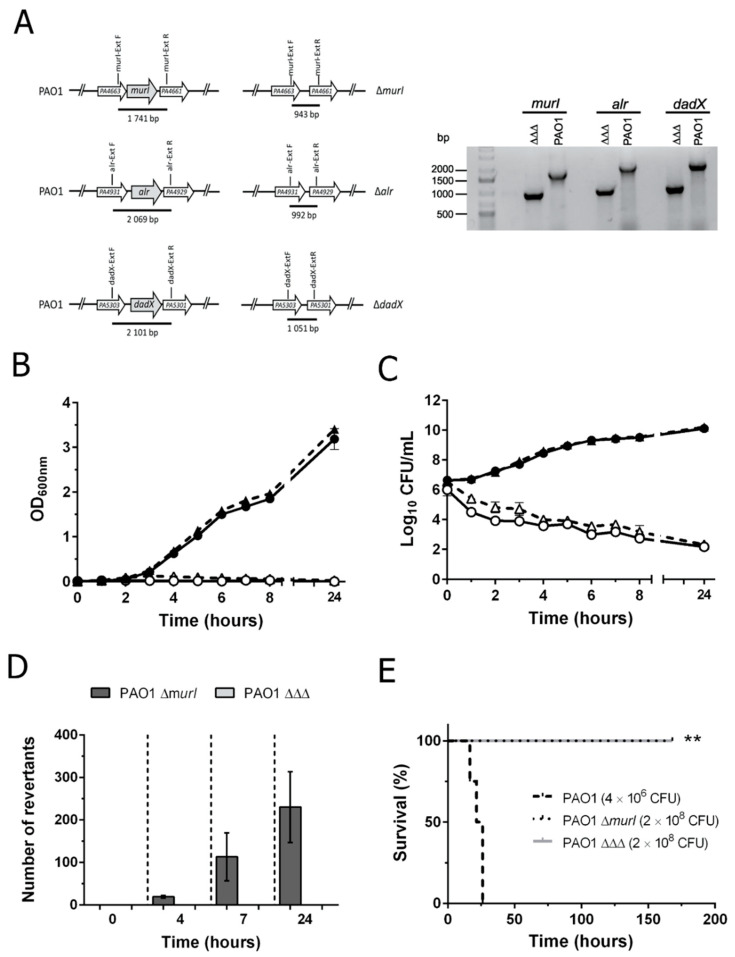
Characterization of PAO1 Δ*murI* Δ*alr* Δ*dadX* (PAO1 ΔΔΔ). (**A**) Confirmation by PCR of *murI, alr* and *dadX* gene deletions in the PAO1 derivative. Schematic representation of the genetic context surrounding *murI* (PA4662), *alr* (PA4930) and *dadX* (PA5302) genes and PCR amplification of *murI*, *alr* and *dadX* regions of PAO1 wild-type and PAO1 ΔΔΔ with specific primers to confirm gene deletions. Growth (**B**) and viability (**C**) curves of PAO1 ΔΔΔ (circles) relative to PAO1 Δ*murI* (triangles) strains in supplemented (solid symbols) and non-supplemented (open symbols) media. (**D**) Phenotypic reversion of PAO1 Δ*murI* and PAO1 ΔΔΔ at different times. Each data point represents the mean number of revertant colonies detected in LB, when D-glutamate or D-glutamate plus D-alanine are not available, in three independent experiments. (**E**) Virulence attenuation of single and double auxotrophic strains compared to the parental strain PAO1. Survival of BALB/c mice (*n* = 4) injected intraperitoneally (IP) with PAO1, PAO1 Δ*murI* or PAO1 ΔΔΔ strains (** *p* = 0.0017, log-rank Mantel–Cox test).

Two alanine racemases genes were identified in the genome sequence of the PAO1 strain: *alr* (PA4930) and *dadX* (PA5302) [9]. Both genes were in-frame deleted from the chromosome of *P. aeruginosa* PAO1 Δ*murI* [7] by an allelic exchange reaction using the pKNG101 vector [11] (Figure 1A) in order to generate a double auxotroph for D-glutamate and D-alanine. The resultant strain, PAO1 ΔΔΔ, required the addition of both D-amino acids for growth (Figure 1B). No visible growth of the double auxotroph was observed in culture media with only D-glutamate or D-alanine. Notably, a decrease of 3-log units in viable counts was detected for PAO1 ΔΔΔ strain after 2 h during incubation in non-supplemented media (Figure 1C). No differences in growth rates or viability were found between the single and double auxotrophic strains cultured in appropriate supplemented LB broth.

### 3.2. P. aeruginosa PAO1 ΔΔΔ Has a More Stable and Safer Auxotrophic Phenotype Than PAO1 ΔmurI

In order to compare the phenotypic stability of both auxotrophic strains and to detect the appearance of prototrophic revertants, cultures were grown in supplemented media and samples were removed at different times for plating on LB agar. For the single auxotroph, PAO1 Δ*murI*, no CFU were observed on a non-supplemented medium for bacteria concentrations equal to (or less than) 1.6 × 10^9^ CFU/mL. To increase the detection threshold, a 500 mL culture of the double auxotroph grown in supplemented media was then plated on media lacking D-glutamate and D-alanine; no PAO1 ΔΔΔ revertant clones arose out of 4.3 × 10^12^ CFU plated under these conditions (Figure 1D). In addition, the stability of the nutritional auxotrophy was also evaluated for 5 days of incubation and again, no revertant colonies for PAO1 ΔΔΔ were found. Thus, the phenotype reversion frequency of the double auxotroph was established as 10^−12^ events, a significantly lower frequency than in PAO1 Δ*murI*. Furthermore, the persistence of the PAO1 ΔΔΔ strain in the environment was much lower than that of the wild-type strain, as no viable bacteria were recovered after 20 days in water (data not shown), similar to reports for the D-glutamate auxotrophic strain [7].

### 3.3. PAO1 ΔΔΔ Strain Is Attenuated in BALB/c Mice

To test the effect of the double auxotrophy for D-glutamate and D-alanine on *P. aeruginosa* virulence and comparison with that exhibited by the single auxotroph [7], a mouse model of systemic infection was used. BALB/c mice were injected with IP with PAO1 wild-type, PAO1 Δ*murI* or PAO1 ΔΔΔ strains, and survival was then monitored for seven days. As shown in Figure 1E, all mice infected with the wild-type strain PAO1 (4 × 10^6^ CFU) succumbed to infection, while all mice inoculated with a 2-log higher dose of the single or double auxotroph (2 × 10^8^ CFU) survived infection. Therefore, the level of attenuation produced by the double auxotrophic strain PAO1 ΔΔΔ is equivalent to that produced by the aforementioned single auxotroph of *P. aeruginosa*. Moreover, both vaccine candidates were less virulent than the wild-type strain PAO1.

### 3.4. PAO1 ΔΔΔ Strain Preserved Immunogenicity and Showed a Similar Level of Protective Efficacy to the Previous Vaccine Prototype, PAO1 ΔmurI, against Acute Lung Infection

**Figure 2:**
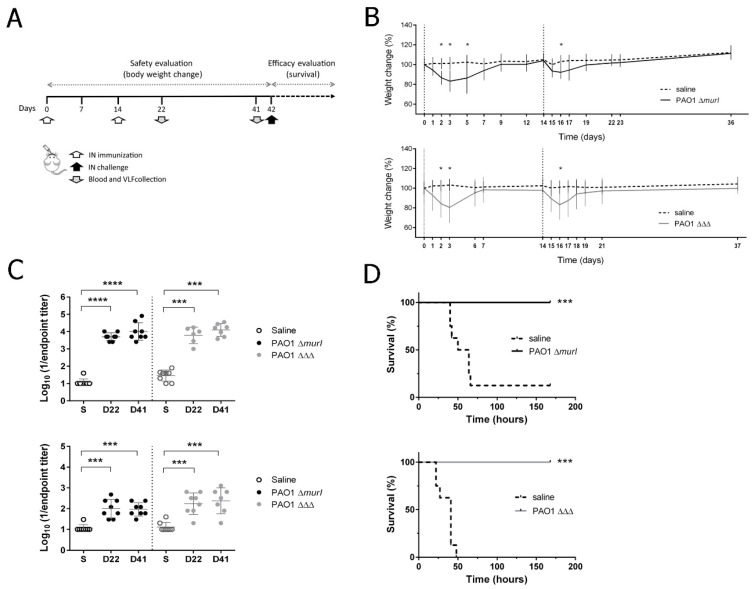
Safety, humoral and mucosal responses, and protection produced after intranasal (IN) inoculation. (**A**) Two-dose vaccine schedule for the IN delivery route. (**B**) Percent of body weight change in BALB/c mice (*n* = 8) after two IN immunizations (days 0 and 14) with PAO1 Δ*murI* (2 × 10^8^ CFU) or PAO1 ΔΔΔ (4 × 10^8^ CFU) or saline administration. Mean ± SD. * *p* < 0.05, unpaired *t*-test (Holm–Sidak method). (**C**) Serum IgG (upper panel) and vaginal IgA (lower panel) endpoint titers in immunized mice with PAO1 Δ*murI* or PAO1 ΔΔΔ produced against PAO1 were determined by ELISA. *** *p* < 0.001, **** *p* < 0.0001, Mann–Whitney test, compared to saline group. S, saline; D, day. (**D**) Protection efficacy of vaccine candidates against acute pneumonia. Four weeks after the last IN immunization, BALB/c mice (*n* = 8) were challenged via the IN route with PA14 (1 × 10^6^ CFU) and survival rates were monitored. *** *p* < 0.001, log-rank Mantel–Cox test, relative to the group administered saline.

To assess the humoral response generated with the auxotrophic vaccine candidates, BALB/c mice were immunized by the IN route with two doses of PAO1 Δ*murI* (2 × 10^8^ CFU) or PAO1 ΔΔΔ (4 × 10^8^ CFU) at a 14-day interval (Figure 2A). Unfortunately, IN administration of both strains at a high vaccine dose (above 10^8^ CFU) had a toxic effect and led to a transient body weight loss in inoculated mice (Figure 2B).

After two inoculations with the auxotrophic strains, significant levels of PAO1-specific antibodies were detected on day 22 in sera (IgG, *p* < 0.0001, Mann–Whitney test) and VLF (IgA, *p* < 0.001, Mann–Whitney test) relative to those detected to the sham-immunized mice; these levels were maintained until day 40 (Figure 2C). Specifically, PAO1 ΔΔΔ elicited a significant increase of about 2-log units in serum IgG and IgA, relative to the sham-immunized mice, whereas the specific-antibody response elicited by PAO1 Δ*murI* was slightly higher for IgG (3-log units) and similar for IgA (Figure 2C).

To establish the protective efficacy against an acute lung infection, groups of immunized mice with each mutant strain and mock groups were challenged by IN inoculation with 1 × 10^6^ CFU of hypervirulent PA14 strain, four weeks after the last booster (on day 42; Figure 2A). Survival was monitored over time. Almost all sham-immunized mice succumbed to the infection within 48–72 h. Conversely, all immunized mice survived and did not show any signs of disease during the observation period (Figure 2D); therefore, the novel vaccine candidate, PAO1 ΔΔΔ, confers similar protection against acute lung infection to that previously described for D-glutamate auxotroph of *P. aeruginosa* [7,8].

## 4. Discussion

Live vaccines elicit cell and antibody-mediated immunity, but they should be sufficiently attenuated by natural selection or genetic engineering to prevent the risk of clinical infection in the vaccinated population or by unintentional release to the environment. Therefore, possible reversion to virulence is one of the main safety concerns of vaccines based on living organisms [13]. We have observed that the vaccine candidate PAO1 Δ*murI* is sporadically able to restore D-glutamate prototrophy when used at high concentrations. Similarly, the D-glutamate auxotrophy of a *murI* mutant of *Vibrio fischeri* was restored by overexpression of the aspartate racemase, RacD [14]. Likewise, an L-aspartate/glutamate racemase from *E. coli* containing only one cysteine residue in the active site, EcL-DER, showed threefold higher racemase activity with L-glutamate than L-aspartate [15]. We speculate that, in a similar manner, a putative aspartate racemase from *P. aeruginosa* may lose its substrate specificity, converting L-glutamate into D-glutamate and therefore, substituting the activity of MurI. Notably, the main safety issue related to the probability of phenotypic reversion to virulence has been successfully addressed in this study. The double D-glutamate and D-alanine auxotroph of *P. aeruginosa* was constructed by deletion of *alr* and *dadX* genes in PAO1 Δ*murI*, blocking the incorporation of both D-amino acids into peptidoglycan. In the present study, we demonstrated that the double auxotroph strain is attenuated, elicits PAO1-specific IgG and IgA antibodies and confers protection against acute pneumonia in mice, as occurs with the single auxotroph [7,8]. Regrettably, an adverse effect was observed with both auxotrophic strains as mice given a high dose of vaccine delivered by the IN route showed transient weight loss. This effect could be due to the endotoxic properties of the bacterial lipopolysaccharide (LPS). The lipid A moiety of the LPS is recognized by Toll-like receptor 4, which activates the production of proinflammatory cytokines and innate immunity, but may have fatal consequences [16,17,18]. In future research, a vaccine strain with a less reactogenic LPS may be engineered. Notwithstanding, the stability and nonreversion to prototrophy in the double auxotrophic mutant of PAO1 support the proposal of this strain as an effective and safer vaccine candidate for the prevention of respiratory *P. aeruginosa* infections.

## 5. Conclusions

We have improved the safety of the previously developed anti-*P. aeruginosa* vaccine candidate with the simultaneous deletion of *alr1*, *dadX* and *murI* genes in PAO1, which resulted in double auxotrophy for D-glutamate and D-alanine and virulence attenuation in a murine sepsis model. The novel live double auxotrophic vaccine, PAO1 ΔΔΔ, retains the fitness, immunogenicity and efficacy of the single auxotrophic strain, providing similar protection against the hypervirulent PA14 lethal pneumonia. The PAO1 ΔΔΔ strain substantially improved the auxotrophic phenotype stability, thereby producing a safer candidate vaccine against respiratory infections caused by *P. aeruginosa*.

## Figures and Tables

**Table 1:** List of primers used in the present study.

Primer Name	Direction	Sequence (5′ to 3′) *	Comments
UP-PA4930(BamHI)-FW	Forward	CGC**GGATCC**GCACCGACAAGGCCGTGTTG	To amplify the upstream region of PA4930 (*alr*)
UP-PA4930(NotI)-RV	Reverse	CCC**GCGGCCGC**GGCATCGGGTCCTGCAAC	
DN-PA4930(NotI)-FW	Forward	CCC**GCGGCCGC**TTCAGGAGATACGCTCCG	To amplify the downstream region of PA4930 (*alr*)
DN-PA4930(ApaI)-RV	Reverse	TTT**GGGCCC**TGGCCCTGG	
UP-PA5302(SalI)-FW	Forward	CTT**GTCGAC**CCCGATCGTCGGCGCC	To amplify the upstream region of PA5302 (*dadX*)
UP-PA5302(NotI)-RV	Reverse	CCC**GCGGCCGC**GGCGACGGGTCTCTCTTC	
DN-PA5302(NotI)-FW	Forward	CCC**GCGGCCGC**GAAAAACTTTCCGAATTC	To amplify the downstream region of PA5302 (*dadX*)
DN-PA5302(ApaI)-RV	Reverse	TTT**GGGCCC**GGCGGGGTC	
PA4662-EXTFW	Forward	GTATCGGCAAGGTGGAGT	To confirm PA4662 (*murI*) gene deletion
PA4662-EXTRV	Reverse	GAATGGCTTGATCGAGTC	
UpKn	Forward	CCCTGGATTTCACTGATGAG	Universal pKNG101 primers flanking the multiple cloning site
RpKn	Reverse	CATATCACAACGTGCGTGGA	
*alr*-EXT F	Forward	GATCATGATCGACTACCT	To confirm PA4930 (*alr*) gene deletion
*alr*-EXT R	Reverse	GATGGAGTTCGCCGAAAG	
*dadX*-EXT F	Forward	CGCAGATCAGTACCGAAG	To confirm PA5302 (*dadX*) gene deletion
*dadX*-EXT R	Reverse	CGTGGTTGAGCATTTCCT	

* The restriction enzyme sites are shown in bold.
